# Atypical Parathyroid Adenoma Complicated with Protracted Hungry Bone Syndrome after Surgery: A Case Report and Literature Review

**DOI:** 10.1155/2015/757951

**Published:** 2015-11-12

**Authors:** Óscar Alfredo Juárez-León, Miguel Ángel Gómez-Sámano, Daniel Cuevas-Ramos, Paloma Almeda-Valdés, Manuel Alejandro López-Flores A La Torre, Alfredo Adolfo Reza-Albarrán, Francisco Javier Gómez-Pérez

**Affiliations:** ^1^Endocrinology and Metabolism Department, Instituto Nacional de Ciencias Médicas y Nutrición Salvador Zubirán, 14080 Mexico City, Mexico; ^2^School of Medicine, Universidad Panamericana, 03920 Mexico City, Mexico

## Abstract

Hungry Bone Syndrome refers to the severe and prolonged hypocalcemia and hypophosphatemia, following parathyroidectomy in patients with hyperparathyroidism. We present the case of an eighteen-year-old woman with a four-year history of hyporexia, polydipsia, weight loss, growth retardation, and poor academic performance. The diagnostic work-up demonstrated primary hyperparathyroidism with hypercalcemia of 13.36 mg/dL, a PTH level of 2551 pg/mL, bone brown tumors, and microcalcifications within pancreas and kidneys. Neck ultrasonography revealed a parathyroid adenoma of 33 × 14 × 14 mm, also identified on ^99^Tc-sestamibi scan. Bone densitometry showed decreased *Z*-Score values (total lumbar *Z*-Score of −4.2). A right hemithyroidectomy and right lower parathyroidectomy were performed. Pathological examination showed an atypical parathyroid adenoma, of 3.8 g of weight and 2.8 cm in diameter. After surgery she developed hypocalcemia with tetany and QTc interval prolongation. The patient required 3 months of oral and intravenous calcium supplementation due to Hungry Bone Syndrome (HBS). After 42 months, she is still under oral calcium. Usually HBS lasts less than 12 months. Therefore we propose the term “Protracted HBS” in patients with particularly long recovery of 1 year. We present a literature review of the diagnosis, pathophysiology, and treatment of HBS.

## 1. Introduction

The Hungry Bone Syndrome (HBS) was first described by Albright and Reifenstein in 1950, in patients with hyperparathyroidism with a severe and prolonged hypocalcemia after parathyroidectomy [1].

Most HBS definitions consider clinical manifestations of hypocalcemia and biochemical variables including hyperparathyroidism requiring surgery, hypophosphatemia, and hypomagnesemia. Some authors denote HBS when patients develop postoperative hypocalcemia (<8.5 mg/dL), a simultaneous inorganic phosphate value of <3.0 mg/dL [2], and hypocalcemia longer than 4 days requiring calcium supplementation, despite optimization of supportive therapy with normal vitamin D levels [3, 4].

Parathyroid hormone (PTH) and calcitriol (1*α*,25[OH]_2_D_3_) regulate calcium and phosphate homeostasis. PTH is secreted in response to hypocalcemia after being sensed by the parathyroid calcium-sensing receptor (CaSR) [5]. PTH receptors are mainly present in kidney and bone tissue [6], and when activated, they increase bone calcium efflux and decrease renal excretion to maintain normal serum calcium concentrations. The HBS pathophysiology begins with elevated PTH production (primary, secondary, or tertiary hyperparathyroidism) which augments bone metabolism and calcium turnover, leading to increased serum calcium levels. Treatment of primary hyperparathyroidism often requires surgical resection of an adenoma, causing a sudden halt in bone turnover. Consequently, a marked depletion of serum circulating calcium, phosphate, and magnesium is seen due to bone remineralization [2].

The most common etiologies associated with HBS are secondary and primary hyperparathyroidism [3, 7, 8]. Other less frequent causes are parathyroid carcinoma, drugs, multiple endocrine neoplasia, and metastatic prostate cancer [9–14].

Here we present a clinical case of an unusual long-lasting HBS developed in a female patient with primary hyperparathyroidism after surgical treatment of an atypical adenoma.

## 2. Case Presentation

An 18-year-old previously healthy woman presented at our institution after a four-year history of hyporexia, polydipsia, weight loss, growth retardation, and poor academic performance. One month prior to presentation, outpatient laboratory analysis revealed increased serum calcium and parathyroid hormone (PTH). She did not have any other significant personal or family history.

She was admitted to our hospital in February 2010. On physical examination her height was 1.40 m and weight was 30 kg. Her vital signs were within normal limits. She presented with a marked cervical kyphosis.

Relevant laboratory results showed hypercalcemia (13.36 mg/dL, normal 8.6–9.9 mg/dL), elevated PTH (2551 pg/mL, normal 12–88 pg/mL), elevated alkaline phosphatase level (4410 IU/L, normal 34–104 IU/L), hypercalciuria (urinary calcium of 213 mg/24 H, normal 100–250 mg/24 H), low creatinine clearance (25.84 mL/min/1.73 m^2^, normal ≥90 mL/min/1.73 m^2^), and low 25[OH]D_3_ circulating level (13 ng/mL, normal 30–100 ng/mL). The laboratory findings at hospital admission and last follow-up visit are summarized in Table 1. Biochemical approach was further completed with prolactin 12.6 ng/mL, FSH 4.4 mIU/mL, LH 17 mIU/mL, estradiol 36.13 pg/mL, T3 1.78 nmol/L, T4 69.33 nmol/L, TSH 1.85 *μ*UI/mL, thyroglobulin 5.7 ng/mL, ACTH 19 pg/mL, and morning cortisol 15.76 *μ*g/dL, all within normal limits.

**Figure 1:**
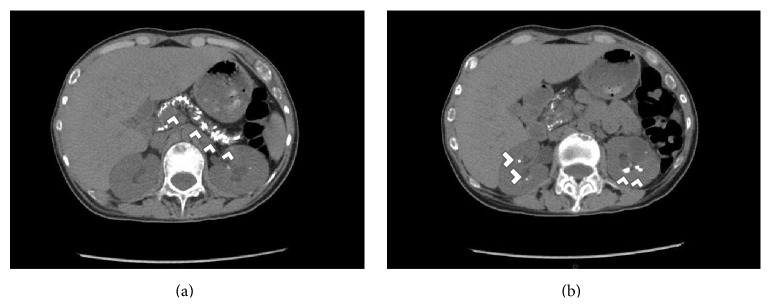
Abdominal computed tomography (CT) on admission. (a) Diffuse pancreatic calcifications; (b) bilateral kidney calcifications on axial computed tomography. Findings are marked with white arrow heads.

**Figure 2:**
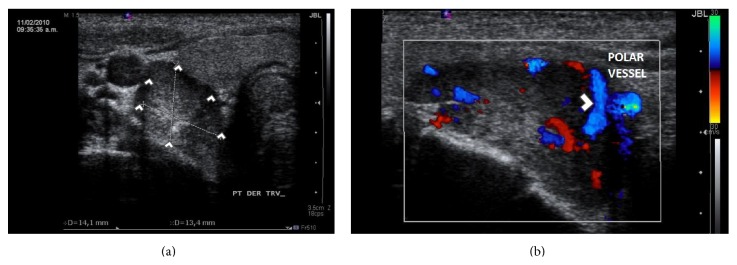
Neck ultrasonography on admission. (a) Large echogenic mass dorsal to the right lobe of the thyroid gland. (b) Doppler effect showing polar vessel finding, present in most adenomas. Findings are marked with white arrow heads.

**Figure 3:**
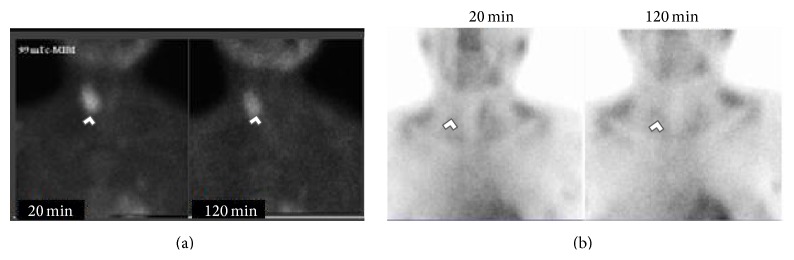
Neck scintigraphies with 25 mCi of ^99^Tc-sestamibi with 0′ and 120′ wash-out sequences. (a) 2010 Admission Scintigraphy. 120′ washout sequence shows residual capitation from right lower thyroid lobe suggesting a parathyroid adenoma. (b) 2013 postparathyroidectomy control scintigraphy. 120′ wash-out sequence shows no apparent residual captation. Findings are marked with white arrow heads.

Skeletal X-rays showed skull with “salt and pepper” lesions, vertebral compression fractures, brown tumors within the left humerus, and profuse calcifications within the pancreas and kidneys (Figure 1). The patient did not refer to abdominal pain, nor any symptomatology related to endocrine and exocrine pancreatic insufficiency. The electrocardiogram was unremarkable. Abdominal ultrasound revealed kidney stones causing bilateral dilation of the renal pelvis. Neck ultrasonography showed microcalcifications within a large echogenic mass, posterior to the right lobe of the thyroid gland of 33 × 14 × 14 mm, Figure 2. A 25 mCi ^99^Tc-sestamibi scintigraphy reported persistence of the radionuclide material at 120 minutes in the right inferior parathyroid gland at the same location of the mass shown in the US, Figure 3. Bone densitometry showed decreased *Z*-Score values (total lumbar *Z*-Score of −4.2), Table 2. Primary hyperparathyroidism secondary to a large parathyroid tumor was diagnosed.

For HBS prophylaxis, before surgery, she received a 400,000 IU of vitamin D2 (Drisdol) (ergocalciferol) and 1 mg of calcitriol. Right hemithyroidectomy with right lower parathyroidectomy was performed. A tumor of 2.8 cm with 3.8 g of weight was resected. Pathological revision reported a focal lesion containing parathyroid tissue; it infiltrated the nodule capsule, without trespassing it, and the diagnosis was consistent with an atypical parathyroid adenoma. Two lymph nodes demonstrated follicular hyperplasia. Despite the administration of vitamin D (50,000 per week) and calcitriol 0.75 mg per day after surgery, the 25-hydroxyvitamin D levels never reached a value above 30. 1,25-dihydroxy vitamin D was not measured.

**Figure 4:**
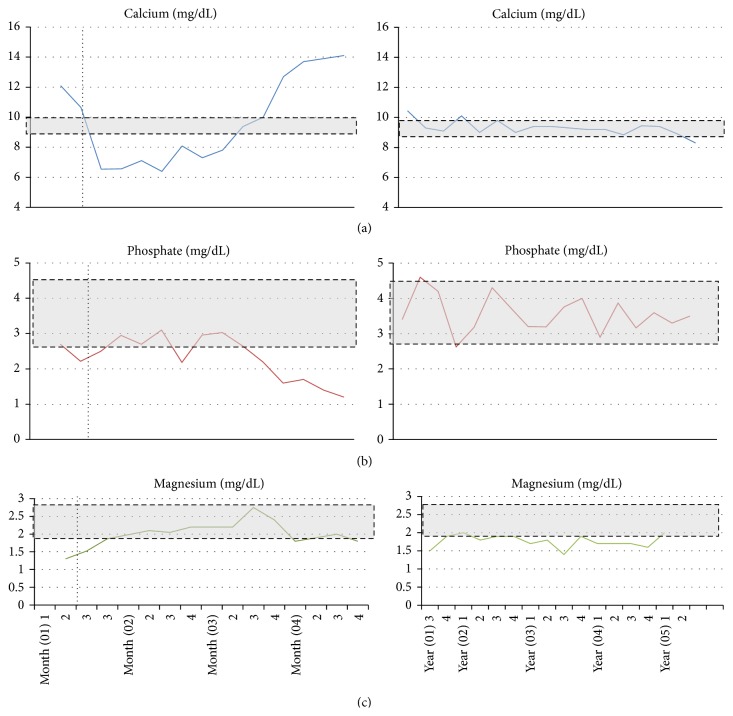
(a) Corrected serum calcium during hospitalization and as outpatient. (b) Serum phosphate values during hospitalization and as outpatient. (c) Serum magnesium values during hospitalization and as outpatient. ^*∗*^Gray area represents reference values. ^*∗∗*^Vertical dotted line represents treatment beginning, which continued beyond last medical assessment at our institution.

Postsurgical laboratory analysis showed PTH values of 31.3 pg/mL, serum calcium of 8.5 mg/dL, phosphate of 1.9 mg/dL, and magnesium of 1.8 mg/dL; on physical examination she presented upper extremity distal contractures, oromandibular dystonia, Chvostek and Trousseau signs, and QTc interval prolongation. PTH levels reached up to 48.6 pg/mL after 1 month, coexisting with hypophosphatemia of 2.7 mg/dL. At last follow-up, PTH serum levels were between 80 and 90 pg/mL, Table 2. She developed a prolonged and severe HBS that required 3 months with oral and intravenous calcium supplementation. The calcium IV infusion was stopped three months later. High PTH levels with hypocalcemia but also hypophosphatemia ruled out hypoparathyroidism. “Protracted” HBS was therefore diagnosed. During her hospitalization she underwent two episodes of lithotripsy as treatment for the kidney stones. Serum calcium, phosphate, and magnesium levels during hospitalization and follow-up are shown in Figure 4.

After 42 months of treatment, bone densitometry scores improved within normal limits, Table 2. As an outpatient since 2010 she has been receiving an average of 1197 mg of oral elemental calcium, 1600 units of vitamin D, and 3 grams of magnesium sulfate per day. She was kept under close medical evaluation as an outpatient. Unfortunately in November 2013 the patient stopped coming to our institution with no clear reason, Table 1.

## 3. Discussion

We described a case of “Protracted” HBS in a female patient following parathyroidectomy for primary hyperparathyroidism caused by an atypical parathyroid adenoma. The primary hyperparathyroidism remained undiagnosed for at least 4 years and was complicated by the occurrence of brown tumors, severe osteoporosis, nephrocalcinosis, and short stature.

Postoperative hypocalcemia following parathyroidectomy could be associated with numerous causes. Some common causes are transient hypoparathyroidism due to extensive surgical removal of parathyroid glands, disruption of blood supply to remnant parathyroid glands, radical neck exploration, and major remineralization of bone such as in HBS [2, 7, 15]. The main features that favor the diagnosis of hypoparathyroidism versus HBS are the extent of surgery, bilateral neck exploration, or prior neck surgery. Also hypoparathyroidism usually presents with hypocalcemia and hyperphosphatemia [2, 16]. HBS is associated with high and long term requirements of calcium and vitamin D supplementation with normal or high PTH levels [2, 7].

The lack of consensus for the definition of HBS hinders the comparison of reported cases, validation of risk factors, determination of severity and prognosis predictors, proposal of prophylaxis regimens, and delineation of treatment goals, as well as resolution criteria [4, 7].

Clinical and laboratory risk factors for HBS are older age, weight and volume of resected parathyroid glands, elevated alkaline phosphatase, evidence of significant bone disease, and blood urea nitrogen [2, 7]. The length of hospitalization has been associated with severity of hypocalcemia [2].

In order to prevent HBS, some authors suggest treatment with bisphosphonates in primary [17] and secondary hyperparathyroidism [18]; however, this approach may delay bone remodeling [19].

The lesion resected from our patient was compatible with atypical adenoma, because it presented capsule invasion without trespassing its boundaries, the rest of the surgical piece was analyzed finding normal thyroid and lymph nodes without invasion. According to the World Health Organization criteria (WHO, 2004) [20] an atypical adenoma refers to large glands with either excess mitotic cells and tumor capsule invasion without exceeding its boundaries, or with marked fibrotic divisions pattern. It is associated with neither spontaneous tumor necrosis, nor vascular invasion to surrounding tissues [21, 22]. These pathology characteristics have been usually associated with a benign course in terms of survival. Parathyroid carcinoma specimens have been associated with the development of HBS [8, 10–12].

Due to the sporadic nature, severity of symptoms, and young age at presentation, genetic abnormalities could be suspected as the cause of this clinical presentation, such as gain of function of protooncogenes (e. gr. cyclinD1/PRAD1), or inactivation of tumor suppressor genes (e. gr. MEN1, multiple endocrine neoplasia 1).

Primary hyperparathyroidism (PHPT) is the most common feature of MEN1 and presents in approximately 90% of MEN1 patients [23, 24]. The PHPT main manifestations reported as part of MEN1 syndrome are earlier age at onset (20 to 25 yrs* versus* 55 yrs), demineralization, and/or recurrent kidney stones [24], findings consistent with the presentation of our patient. Normal laboratory analysis ruled out functional pituitary adenoma at this time; however, she will continue to be under close observation.

Since the patient did not refer to any symptomatology for the suspicion of a functional pancreatic/gastrointestinal (GI) adenoma, no additional biochemical analyses were carried out. No apparent pancreatic tumors were found on the abdominal CT.

Clinical manifestations of hypocalcemia in HBS range from relative benign symptoms such as weakness, headache, paresthesias, ileus, malabsorption, and muscle cramps to life threatening features such as arrhythmias, seizures, laryngeal stridor, tetany, and overt severe heart failure [4, 7, 25]. Our patient presented mainly musculoskeletal manifestations.

In patients with HBS, serum electrolytes, such as calcium, phosphate, and magnesium, should be cautiously monitored over the first postoperative hours and days, as severe electrolytes disturbances may develop [2, 3]. During hospitalization the patient required up to 1289 mg of elemental calcium IV per day or 42.9 mg/kg of elemental calcium IV. Treatment of hypocalcemia is based on oral and intravenous calcium replacement. Reported daily requirements of calcium in patients with severe hypocalcemia range from 6 to 16 g of elemental calcium per day [7, 10, 26–28].

The preferred calcium administration route depends on signs and symptoms severity, promptness of the onset of manifestations, and serum calcium levels [29, 30]. Oral calcium supplementation could be reasonably used in patients with mild symptoms and serum calcium concentrations greater than 7.5 mg/dL. Intravenous treatment is required for patients with calcium below this level or prolonged QTc interval on electrocardiogram and may be necessary for those cases who are currently unable to swallow or absorb oral calcium [30–32].

Intravenous calcium gluconate is preferred over calcium chloride due to its lower association with local irritation. For acute hypocalcemia management, one or two 10 mL ampoules of 10% calcium gluconate (10 mL of a 10% solution = 93 mg elemental Ca or 1 ampule) should be diluted in 50–100 mL of 5% dextrose or saline in order to infuse it over 10 minutes [10, 31, 33]. After the patient is stable, a calcium infusion is continued adding 10 ampoules of calcium gluconate (100 mL of 10% calcium gluconate) to 1000 mL normal saline or 5% dextrose making up a solution containing 1 mg/mL of elemental calcium [33]. Typical patient requirements are 0.5 to 1.5 mg/kg of elemental calcium per hour [30]. Throughout hospitalization the patient required up to 43,113.6 mg of elemental calcium orally per day (equivalent to 108 tablets of 1 g of calcium carbonate; each tablet contains approximately 400 mg of elemental calcium).

In HBS, once the hyperparathyroid state is resolved, it is important to assess serum magnesium and phosphate circulating concentrations. Significant hypomagnesaemia and hypophosphatemia may develop and perpetuate hypocalcemia [34, 35]. Even though mild hypomagnesaemia habitually is asymptomatic, chronic deficiency may be associated with diverse comorbid situations, such as arrhythmias, hypertension, and an increase in progression of kidney disease [34]. Magnesium levels after parathyroidectomy may decrease secondary to increased bone mineralization, causing a lower PTH secretion and a relative tissue specific resistance, increasing risk of severe hypocalcemia. Therefore, magnesium should be also supplemented [36].

Hypophosphatemia in HBS is probably due to an increase in bone uptake to facilitate matrix remineralization [6]. The administration of phosphate in patients with HBS is generally avoided because phosphate can precipitate with calcium, decreasing even further the circulating calcium concentration. Phosphate administration is reserved to those patients with less than 1.0 mg/dL and severe symptoms such as muscle weakness or heart failure [5]. Phosphate availability increases secondary to the intestinal action of vitamin D.

HBS duration has been defined as the time taken to remineralize the skeleton and/or cessation of additional calcium supplementation [7], evidenced by normalization of bone turnover markers, healing of radiological features of osteitis fibrosa cystica and brown tumours, and significant gain in bone mass. Due to the lack of consensus on definitions for HBS length, we take into account two of the most cited terms found in HBS related literature: bone mineral density (BMD) recovery and calcium supplementation length.

Considering HBS resolution with BMD normalization criteria, HBS lasted from 4.5 to 16 months with a median of 10 months [2, 13, 37–40] (Table 3). Calcium replenishment has been reported throughout 0.5 months to 12 months, with a median of 5.1 months. [3, 10, 13, 26, 41, 42]. Using this information, HBS lasts between 10 and 12 months. We therefore propose the term of “Protracted Hungry Bone Syndrome” to refer to those cases with a particularly long recovery taking more than one year.

In a case series of patients with HBS, parathyroidectomy improved femoral neck bone mineral density (BMD) scores from 35 to 131% in 1 year after surgery, considering basal BMD values ranging from 0.234 to 0.564 g/cm^2^, and 1 year follow-up values of 0.541–0.942 g/cm^2^ [43]. The significant recovery from baseline pathologic findings to the value above normal found in our patient is consistent with previously reported BMD recovery (Table 3). Bone densitometries were only done at hospital admission and three years after surgery as outpatient (Table 2). The factors that contribute to this impressive BMD improvement in our case were the resolution of primary hyperparathyroidism and the high amounts of calcium and vitamin D administered for over 42 months to treat the HBS.

To the best of our knowledge, the case presented here is one of the longest HBS reported. In spite of the HBS criteria described that requires recovery of bone remineralization, we believe that our patient still has HBS, based on the fact that she continues with oral calcium and magnesium supplementation to maintain normal serum levels, normal circulating vitamin D, and normal high PTH levels.

## 4. Conclusion

HBS is a consequence of hyperparathyroidism following parathyroidectomy. It is an infrequent cause of hypocalcemia, hypophosphatemia, and hypomagnesemia that requires adequate therapy to avoid complications in the acute and chronic scenarios. We propose the term of “Protracted Hungry Bone Syndrome” to refer to those cases with a particularly prolonged course of recovery that takes more than one year.

## Figures and Tables

**Table 1:** Laboratory findings at hospital admission and at last outpatient follow-up visit.

	Hospitalization [February 2010]	Follow-up [November 2013]	Reference values
Serum calcium [mg/dL]	12.1	8.9	8.6–9.9
Corrected serum calcium [mg/dL]^*∗*^	12.6	7.9	
Serum phosphate [mg/dL]	2.7	3	2.7–4.5
Serum magnesium [mg/dL]	1.3	2	1.9–2.7
Serum albumin [g/dL]	3.4	5.2	3.5–5.7
Serum creatinine [mg/dL]	4.83	1.05	0.42–1.09
Creatinine clearance [ml/min/1.73 m^2^]	12	76	≥90
PTH [pg/mL]	2551	91.7	12–88
25-OH vitamin D [ng/mL]	13	25.9	30–100
Alkaline phosphatase [IU/L]	4410	126	34–104
Urinary calcium [mg/day]	213	22	100–300
Urinary phosphorus [mg/day]	413	524	<1000
Prolactin [ng/mL]	12.6	—	2.64–13.13
FSH [mIU/mL]	4.4	—	3.85–8.78^(follicular phase)^
LH [mIU/mL]	17	—	2.12–10.89^(follicular phase)^
Estradiol [pg/mL]	36.13	—	12–40^(follicular phase)^
T3 [nmol/L]	1.78	—	0.64–1.81
T4 [nmol/L]	69.33	—	66–181
TSH [*µ*UI/mL]	1.85	—	0.3–5
Thyroglobulin [ng/mL]	5.7	—	0–36.8
ACTH [pg/mL]	19	—	10–100
Morning cortisol [*µ*g/dL]	15.76		6.7–22.6

PTH parathyroid hormone.

^*∗*^Corrected calcium with albumin using the following formula: Ca^2+^_corrected_ = Ca^2+^_measured_ + 0.8 × [4 − albumin_measured_].

**Table 2:** Densitometry values on admission and at the last follow-up visit as an outpatient. The most affected segment is presented.^*∗*^

	Hospitalization		Follow-up	
	[February 2010]		[November 2013]	
Lumbar BMD	L1	Total	L1	Total

[g/cm^2^]	0.53	0.551	1.061	1.07
*Z*-Score	—	−4.2	1.4	0.4
*T* score	−3.6	−4.5	1.2	0.2

Hip BMD	Neck	Total	Neck	Total

[g/cm^2^]	0.41	0.481	0.939	0.998
*Z*-Score^*∗*^	—	—	0.6	0.4
*T* score	−3.9	−3.6	0.5	0.3

Osteoporosis is diagnosed in young adults when both *Z*-score <−2.0 and fractures are present.

^*∗*^Due to the age of presentation, baseline *Z*-scores could not be obtained with the equipment used in our patient.

**Table 3:** 

Time to reach normal bone density values	Time required of calcium replenishment	References
4.5 months	—	[2]
—	5 months	[41]
8 months	—	[37]
—	5.2 months	[26]
—	6 months	[10]
16 months	3 months	[13]
	0.5 months	[3]
12 months (i) 8 months (ii) 12 months (iii) 12 months		[7] [38] [39] [40]
—	12 months	[42]

Median (months): 10	Median (months): 5.1	—
